# Mitophagy in inflammatory bowel disease: regulatory mechanisms and therapeutic potential

**DOI:** 10.3389/fcell.2026.1804813

**Published:** 2026-05-14

**Authors:** Sixuan Chen, Jing Guan, Xia Pang, Xi Chen, Min Li, Huan Wang, Fei Mao

**Affiliations:** 1 Department of Laboratory Medicine, School of Medicine, Jiangsu University, Zhenjiang, China; 2 The People’s Hospital of Danyang, Affiliated Danyang Hospital of Nantong University, Zhenjiang, China; 3 Department of Pediatrics, the Affiliated Jiangning Hospital of Nanjing Medical University, Nanjing, China; 4 Department of Laboratory Medicine, the Affiliated Jiangning Hospital of Nanjing Medical University, Nanjing, China

**Keywords:** genetic susceptibility, immune cells, inflammatory bowel disease, intestinal barrier, mitophagy

## Abstract

Inflammatory bowel disease (IBD), encompassing Crohn’s disease (CD) and ulcerative colitis (UC), is a chronic inflammatory disorder of the gastrointestinal tract driven by complex interactions among genetic susceptibility, barrier dysfunction, and immune dysregulation. Mitophagy, the selective autophagic clearance of damaged mitochondria, has emerged as a key regulator of intestinal homeostasis and immune balance. Impaired mitophagy compromises epithelial barrier integrity, amplifies inflammatory signaling, and promotes disease progression. This review summarizes the molecular mechanisms of mitophagy, examines its involvement in IBD pathogenesis across intestinal epithelial and immune cell compartments, and highlights mitophagy-modulating compounds that may inform the development of novel therapeutic strategies.

## 1 Introduction

Inflammatory bowel disease (IBD) is a chronic, recurrent inflammatory condition of the gastrointestinal tract, primarily comprising ulcerative colitis (UC) and Crohn’s disease (CD). In recent years, the global incidence of IBD has continued to rise, with particularly marked increases observed among the adolescent population (M’Koma, 2013; Uhlig et al., 2021). The pathogenesis of IBD is complex and multifactorial, involving genetic susceptibility, disruption of the intestinal mucosal barrier, dysbiosis of the gut microbiota, and dysregulated immune activation; however, its precise mechanisms remain unclear (Danese and Fiocchi, 2006; Zhang and Li, 2014). Conventional immunomodulatory drug therapies, such as glucocorticoids and immunosuppressants, are often associated with significant adverse effects that limit their long-term use. Consequently, attention has increasingly shifted toward identifying novel therapeutic targets and pathways (Bruscoli et al., 2021).

Mitochondria are central regulators of cellular homeostasis, functioning as the primary source of adenosine triphosphate (ATP) through oxidative phosphorylation (OXPHOS) and as key signaling hubs controlling intracellular calcium (Ca^2+^), reactive oxygen species (OS), and cyclic adenosine monophosphate (cAMP) levels (Brookes et al., 2004; Boyman et al., 2020). Under stress or pathological conditions, mitochondria are particularly susceptible to damage, leading to functional impairment and disruption of cellular homeostasis. Their efficient removal via mitophagy, a mitochondrial quality-control mechanism that delivers dysfunctional organelles to lysosomes for degradation, is therefore essential for maintaining cellular and tissue homeostasis. Mitophagy, a specialized form of selective autophagy, targets damaged or excess mitochondria for lysosomal degradation and serves as a critical mitochondrial quality-control mechanism (Palikaras et al., 2018).

Emerging evidence indicates that mitophagy plays an important role in the pathophysiology of IBD. Genetic studies have identified autophagy- and mitophagy-related susceptibility genes, including autophagy-related protein 16-like 1 (*ATG16L1*), nucleotide-binding oligomerization domain-containing protein 2 (*NOD2*), and immune-related GTPase family M (*IRGM*), as contributors to IBD development (Li and Law, 2022). Defective mitophagy compromises mitochondrial energy supply in intestinal epithelial cells and disrupts tight junction integrity, leading to impaired barrier function and increased pathogen translocation (JanssenDuijghuijsen et al., 2017; Tian et al., 2021). In addition, mitophagy regulates both innate and adaptive immune responses, influencing macrophage-derived cytokine release and dendritic cell antigen presentation (Nakahira et al., 2011; Lavoie et al., 2019). Dysregulated mitophagy results in the accumulation of mitochondrial DNA (mtDNA) and reactive oxygen species (ROS), which activate the NOD-like receptor family pyrin domain-containing 3 (NLRP3) inflammasome and nuclear factor kappa B (NF-κB) signaling pathways, thereby promoting excessive production of pro-inflammatory cytokines such as interleukin-1β (IL-1β) and tumor necrosis factor-α (TNF-α) and driving aberrant T helper 1(Th1) and T helper 17 (Th17) immune responses (Nakahira et al., 2011; Geremia et al., 2014). Collectively, impaired mitophagy contributes to intestinal epithelial dysfunction, immune imbalance, and microbial dysbiosis, ultimately exacerbating intestinal inflammation.

Although IBD encompasses both CD and UC, the cellular mechanisms underlying these conditions differ in several aspects. For example, Paneth cells, which are abundant in the small intestine, play a prominent role in CD pathogenesis, whereas UC primarily affects the colon and is more closely associated with epithelial barrier disruption and mucosal immune dysregulation. Nevertheless, mitochondrial dysfunction and impaired autophagy have been reported in both CD and UC, suggesting that mitochondrial quality-control mechanisms, such as mitophagy, may represent shared pathogenic processes across the IBD spectrum. Therefore, while several mechanistic studies discussed in this review derive from CD-related models, particularly those involving Paneth cell biology, evidence from UC studies is also included to provide a comprehensive overview of mitophagy in intestinal inflammation.

Given the central role of mitophagy in maintaining gut homeostasis, therapeutic strategies targeting this process may offer promising avenues for IBD treatment. This review summarizes the molecular mechanisms of mitophagy and discusses its regulatory roles in intestinal cells and gut function, providing insights into the development of novel mitophagy-based therapeutic approaches for IBD.

## 2 Molecular mechanisms of mitophagy

### 2.1 Ubiquitin-dependent pathways

**FIGURE 1:**
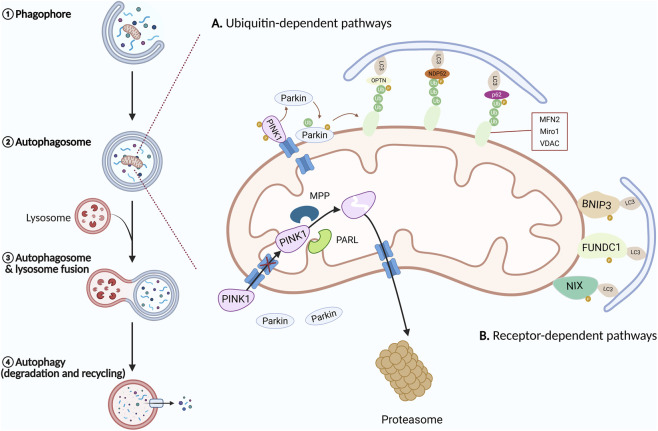
Mechanism of mitophagy. When cells undergo autophagy, there are four main steps: ① initiation of the phagophore membrane that engulfs organelles; ② formation of the autophagosome; ③ fusion of the autophagosome with the lysosome to form the autolysosome; ④ degradation and recycling of cellular components. **(A)** PINK1/Parkin-mediated Mitophagy. **(B)** Receptor-mediated mitophagy. PINK1, PTEN-induced kinase 1; Parkin, Parkin RBR E3 ubiquitin protein ligase; MPP, Mitochondrial processing peptidase; PARL, Presenilin associated rhomboid like; Ub, Ubiquitin; OPTN, Optineurin; NDP52, Nuclear dot protein 52; p62, Sequestosome 1; LC3, Microtubule-associated protein 1 light chain 3; Mfn2, Mitofusin2; Miro1, Mitochondrial Rho GTPase 1; VDAC, Voltage-dependent anion channel; BNIP3, BCL2 interacting protein 3; NIX, BCL2/adenovirus E1B 19-kDa-interacting protein 3-like; FUNDC1,FUN14 domain-containing 1.

In mammals, mitophagy is predominantly regulated by the PTEN-induced kinase 1 (PINK1) and the E3 ubiquitin ligase Parkin pathways, which represent the best-characterized mechanisms for the clearance of damaged mitochondria (Figure 1). Under physiological conditions, PINK1 is imported into mitochondria via the translocase of the outer and inner mitochondrial membrane complexes and is subsequently cleaved and degraded, preventing its accumulation and mitophagy activation (Jin et al., 2010; Pickles et al., 2018; Vizziello et al., 2021). This ensures that Parkin remains inactive under basal conditions.

Upon mitochondrial damage or loss of membrane potential, PINK1 import is blocked, leading to its stabilization on the outer mitochondrial membrane (OMM). Stabilized PINK1 recruits and activates Parkin through phosphorylation events involving ubiquitin and Parkin itself, thereby relieving Parkin autoinhibition and enabling its E3 ubiquitin ligase activity (Narendra et al., 2010; Kane et al., 2014; Gladkova et al., 2018). Activated Parkin ubiquitinates multiple OMM proteins, including mitofusins and voltage-dependent anion channels, thereby labeling damaged mitochondria for autophagic degradation (Sarraf et al., 2013). These ubiquitin tags serve as recruitment signals for autophagy adaptor proteins such as p62 and optineurin, which link ubiquitinated mitochondria to LC3-positive autophagosomes, ultimately leading to lysosomal degradation of dysfunctional mitochondria (Heo et al., 2015; Lazarou et al., 2015). This process is counterregulated by deubiquitinating enzymes such as USP30, which modulate mitophagy intensity (Ordureau et al., 2014).

Importantly, PINK1/Parkin-mediated mitophagy also contributes to intestinal homeostasis. Activation of this pathway protects intestinal epithelial cells from mitochondrial dysfunction, excessive ROS production, and apoptosis, thereby preserving epithelial barrier integrity (Cao et al., 2021). In contrast, impaired PINK1/Parkin signaling leads to accumulation of damaged mitochondria, enhanced inflammasome activation, and exacerbation of intestinal inflammation (Wu S. et al., 2024). Reduced expression of PINK1 and Parkin has been observed in inflamed intestinal tissues, supporting the role of defective mitochondrial quality control in IBD pathogenesis (Chen et al., 2025).

### 2.2 Ubiquitin-independent receptor-mediated pathways

In addition to ubiquitin-dependent mechanisms, mitophagy can be initiated through receptor-mediated pathways involving outer mitochondrial membrane (OMM)-localized proteins that directly bind microtubule-associated protein 1 light chain 3 (LC3) via LC3-interacting region (LIR) motifs, independent of ubiquitination. Key mitophagy receptors include the BCL2-interacting protein 3 (BNIP3) receptor, the BCL2/adenovirus E1B 19-kDa-interacting protein 3-like (BNIP3L/NIX) receptor, and the FUN14 domain-containing 1 (FUNDC1) receptor (Novak et al., 2010; Kuang et al., 2016; Yao et al., 2024). BNIP3 and NIX are BH3-only members of the BCL-2 family that share approximately 56% sequence homology. They are both anchored to the outer mitochondrial membrane (OMM) via a C-terminal transmembrane domain (TMD), exposing their N-terminal LIR motif to the cytoplasm for direct binding to the LC3 protein on autophagosomes (Matsushima et al., 1998; Hanna et al., 2012). Under normal conditions, BNIP3 is found in the cytoplasm as an inactive monomer. When exposed to stress, such as hypoxia, it forms stable homodimers via its TMD and anchors to the OMM (Kubli et al., 2008; Onishi et al., 2021). Phosphorylation of Ser17 and Ser24 within the BNIP3 LIR motif enhances its interaction with LC3, while hypoxia-inducible factor-1α (HIF-1α) acts as a major upstream regulator of BNIP3 and NIX expression (Zhu et al., 2013; Sulkshane et al., 2021).

NIX plays a critical role in selective mitochondrial clearance during erythrocyte maturation, as evidenced by defective mitophagy and anemia observed in NIX-deficient mice (Schweers et al., 2007; Sandoval et al., 2008). Phosphorylation of residues within the NIX LIR motif enhances LC3 binding, whereas mutations disrupting NIX dimerization impair mitophagy (Novak et al., 2010; Rogov et al., 2017; Marinković et al., 2021). Although NIX expression is upregulated upon BNIP3 loss, this compensation is insufficient to fully restore mitophagy activity, indicating partially non-redundant functions (Shi et al., 2014). Beyond their independent roles, BNIP3 and NIX interact with the PINK1/Parkin-mediated mitophagy pathway. BNIP3 stabilizes PINK1 on the OMM by inhibiting its proteolytic cleavage, thereby facilitating Parkin recruitment (Zhang et al., 2016). Conversely, Parkin-mediated ubiquitination of NIX promotes recruitment of neighbor of BRCA1 gene 1 (*NBR1*) and enhances mitophagy, while NIX further facilitates mitochondrial depolarization and Parkin accumulation under stress conditions (Ding et al., 2010; Gao et al., 2015). These findings highlight extensive crosstalk between receptor- and ubiquitin-dependent mitophagy pathways.

FUN14 domain-containing protein 1 (FUNDC1) is another essential OMM mitophagy receptor characterized by an N-terminal LIR motif, a transmembrane domain, and a C-terminal tail (Zhang, 2021). FUNDC1-mediated mitophagy is tightly regulated by reversible phosphorylation within its LIR motif. Under basal conditions, phosphorylation of Tyr18 by Src family kinase (Src) and Ser13 by casein kinase 2 (CK2) suppresses LC3 binding (Liu et al., 2012; Chen et al., 2014). During hypoxia or mitochondrial depolarization, Src inactivation and phosphoglycerate mutase family member 5 (PGAM5)-mediated dephosphorylation enhance FUNDC1-LC3 interaction, promoting mitophagosome formation (Liu et al., 2012; Chen et al., 2014; Wu et al., 2014). FUNDC1 also coordinates mitochondrial dynamics by forming mitochondria-endoplasmic reticulum contact sites and recruiting dynamin-related protein 1 (DRP1) to regulate mitochondrial fission, thereby integrating mitochondrial remodeling with selective autophagic clearance (Chen et al., 2016; Wu et al., 2016). Taken together, receptor-mediated mitophagy provides an alternative and complementary mechanism for mitochondrial quality control, particularly under hypoxic or inflammatory stress, emphasizing the coordinated regulation of mitophagy pathways in maintaining cellular and tissue homeostasis.

Although receptor-mediated mitophagy pathways have been extensively characterized in other physiological contexts, emerging evidence suggests that receptors such as BNIP3 and NIX also contribute to intestinal homeostasis and inflammation. In the intestinal epithelium, NIX-mediated mitophagy has been implicated in regulating mitochondrial stress responses and preventing excessive apoptosis under inflammatory conditions (Vincent et al., 2020). Studies of experimental intestinal inflammation indicate that increased mitochondrial ROS can promote interaction between NIX and LC3, thereby initiating mitophagy to limit epithelial damage (Alula et al., 2023). Furthermore, NIX has been reported to interact with the PINK1/Parkin pathway, suggesting coordinated regulation of mitochondrial quality control during intestinal stress (Mentrup et al., 2024). These observations highlight the potential relevance of receptor-mediated mitophagy pathways in the pathogenesis of IBD.

### 2.3 Potential risks of excessive mitophagy

While mitophagy is essential for eliminating damaged mitochondria and maintaining cellular homeostasis, excessive or dysregulated mitophagy can have harmful consequences. A balanced relationship between mitochondrial biogenesis and mitophagy is fundamental for maintaining mitochondrial integrity and cellular function; disruption of this equilibrium, whether through insufficient or excessive mitochondrial turnover, can compromise cellular fitness (Markaki et al., 2018). Mitophagy primarily functions as a protective mitochondrial quality control mechanism rather than a direct inducer of cell death; however, adverse outcomes may arise when mitochondrial removal outpaces the cell’s ability to restore mitochondrial populations through biogenesis (Tang et al., 2025).

One important risk of excessive mitophagy is the development of bioenergetic deficits resulting from mitochondrial clearance. When mitochondrial degradation exceeds replacement capacity, cellular ATP production and metabolic function can be severely impaired. Evidence supporting this concept comes from mutations in FBXL4, which trigger excessive BNIP3/BNIP3L-mediated mitophagy and lead to mitochondrial DNA depletion syndrome. This disorder is characterized by markedly reduced mitochondrial DNA copy number, impaired oxidative phosphorylation, and progressive organ dysfunction (Chen et al., 2023). Similarly, overexpression of Parkin, a central regulator of mitophagy, resulted in rapid development of cardiac hypertrophy, pulmonary edema, and ventricular dysfunction under hemodynamic stress, demonstrating that excessive activation of mitophagy pathways can produce detrimental physiological effects (Shires et al., 2016). Together, these findings highlight the importance of maintaining a coordinated balance between mitochondrial degradation and biogenesis to preserve cellular energy homeostasis.

Moreover, within intestinal epithelial cells (IECs), excessive mitophagy may aggravate epithelial barrier dysfunction by limiting the mitochondrial energy supply required for epithelial renewal and tight junction maintenance. Mitochondrial dysfunction has been widely recognized as a key contributor to IBD pathogenesis, as epithelial barrier integrity depends on adequate energy availability to sustain epithelial turnover and intercellular junction stability (Chernyavskij et al., 2023). Under oxidative stress conditions, the AMPK–PINK1/Parkin signaling pathway normally promotes protective mitophagy that helps maintain epithelial barrier integrity. Inhibition of this pathway using mdivi-1 significantly reduces transepithelial electrical resistance (TER) and increases epithelial permeability, demonstrating that appropriately regulated mitophagy supports barrier function (Cao et al., 2021). However, if mitophagic activity becomes excessive and depletes functional mitochondria beyond the cell’s capacity for replenishment, ATP production may decline, ultimately weakening tight junction maintenance and barrier stability. In addition, altered mitochondrial dynamics have been observed in IECs from patients with IBD, including reduced expression of the mitochondrial fusion protein OPA1 and increased mitochondrial fragmentation, which can promote epithelial progenitor cell death and sustain chronic intestinal inflammation (Bao et al., 2025). During inflammatory stress, mitochondrial architecture in IECs becomes structurally abnormal, with disrupted cristae and elevated DRP1-mediated mitochondrial fission, promoting metabolic reprogramming toward glycolysis and further impairing epithelial barrier function (Astorga et al., 2022). In such conditions, excessive mitophagy could further reduce the already compromised mitochondrial pool, potentially creating a cycle of energy deficiency and barrier deterioration.

Mitophagy also plays complex roles in T cell biology. During memory CD8^+^ T cell formation, Parkin and NIX coordinate mitochondrial remodeling to maintain cellular survival, with Parkin limiting VDAC1-dependent apoptosis and NIX preventing ferroptosis associated with metabolic dysfunction resulting from defective mitophagy (Franco et al., 2023). Conversely, insufficient mitophagy in regulatory T cells (Tregs) during autoimmune responses can disrupt mitochondrial membrane potential, increase mitochondrial ROS generation, elevate metabolic demand, and ultimately impair their immunosuppressive capacity (Lazari et al., 2017). At the same time, excessive mitophagy may also trigger cell death pathways, such as apoptosis or pyroptosis, through prolonged mitochondrial depletion (Shi et al., 2025). For example, when PINK1–Parkin-mediated degradation of the outer mitochondrial membrane occurs without efficient autophagic engulfment, cytochrome c can be released through a BAX/BAK-independent mechanism, activating APAF1- and caspase-9-dependent apoptosis (Quarato et al., 2023). These findings suggest that incomplete or excessive mitophagy may paradoxically promote cell death rather than prevent it.

From a therapeutic perspective, these observations highlight the importance of carefully regulating mitophagy levels when designing treatments for IBD. Excessive stimulation of mitophagy could lead to adverse effects, such as energy depletion or immune suppression. Indeed, while mitophagy deficiency can sensitize β-cells to inflammatory stress, leading to the accumulation of dysfunctional mitochondria and increased cell death (Sidarala et al., 2020), excessive mitophagy may result in immunoparalysis or epithelial barrier dysfunction. Emerging therapeutic compounds such as urolithin A have shown promise by enhancing mitochondrial turnover in inflamed intestinal epithelium; however, their effects must be carefully evaluated to ensure that mitophagy is stimulated without causing excessive mitochondrial loss and bioenergetic imbalance (Mentrup et al., 2024). Consequently, future therapeutic strategies should focus on restoring the dynamic balance between mitophagy and mitochondrial biogenesis rather than simply promoting or inhibiting mitophagic activity.

## 3 Genetic variants associated with IBD and autophagy

Genome-wide association studies (GWAS) provide a powerful framework for elucidating the contribution of common genetic variants to disease susceptibility. With the development of genome-wide single-nucleotide polymorphism (SNP) arrays, over 300 genetic loci linked to IBD have been identified to date, and CD shows a stronger heritability component than UC (Ramos and Papadakis, 2019; Hume et al., 2025). Notably, several IBD susceptibility genes converge on autophagy-related pathways, including nucleotide-binding oligomerization domain-containing protein 2 (*NOD2*), autophagy-related protein 16-like 1 (*ATG16L1*), and immunity-related GTPase family M (*IRGM*), underscoring the importance of autophagy and mitochondrial quality control in disease pathogenesis.

### 3.1 NOD2

Nucleotide-binding oligomerisation domain-containing protein 2 (*NOD2*) was the first IBD susceptibility gene identified through GWAS*,* highlighting innate immune dysregulation as a central pathogenic mechanism in IBD (Stappenbeck et al., 2011). *NOD2* encodes a cytoplasmic pattern-recognition receptor predominantly expressed in Paneth cells, dendritic cells, macrophages, and intestinal epithelial cells (Baxt and Xavier, 2015; Ramos and Papadakis, 2019). Disease-associated *NOD2* variants, particularly the frameshift mutation Leu1007fs, are associated with reduced α-defensin secretion in Paneth cells and impaired recognition of bacterial peptidoglycans such as muramyl dipeptide (MDP). These defects compromise antimicrobial defense and lead to aberrant activation of NF-κB signaling, thereby promoting intestinal dysbiosis and chronic inflammatory responses (Philpott et al., 2014; Ramos and Papadakis, 2019). Among all known IBD-associated variants, *NOD2* mutations confer the highest genetic risk, increasing susceptibility to CD by approximately 3- to 20-fold (Ismailova and White, 2022). Beyond its role in microbial sensing, *NOD2* directly regulates autophagy by recruiting *ATG16L1* to the plasma membrane at sites of bacterial entry, thereby linking innate immune recognition to autophagosome initiation and intracellular pathogen clearance.

### 3.2 ATG16L1

Autophagy-related protein 16-like 1 (*ATG16L1*) is a central component of the autophagy machinery, and its missense variant Thr300Ala (T300A) is one of the strongest genetic risk factors for CD (Wang et al., 2018). This variant is associated with abnormal Paneth cell granule secretion, increased endoplasmic reticulum stress, and impaired intestinal epithelial barrier function (Deuring et al., 2014; Wang et al., 2018). Mechanistically, T300A reduces *ATG16L1* stability, thereby impairing autophagic capacity, partly by increasing susceptibility to proteolytic cleavage (Lassen et al., 2014; Murthy et al., 2014). Beyond protein stability, structural changes in the T300A variant also affect functional domain interactions, particularly within the WD40 domain, thereby disrupting binding with key autophagy regulators such as WIPI2b and impairing selective autophagy processes (Boada-Romero et al., 2016). This results in altered autophagic cargo selection and reduced bacterial clearance efficiency during infection. In addition, proteomic analyses have shown that the T300A variant alters both the abundance and composition of autophagosomal cargo, particularly under microbial stress conditions, thereby contributing to impaired host defence (Andromaque et al., 2026).

Functionally, *ATG16L1* operates downstream of *NOD2*. Upon bacterial invasion, *NOD2* recruits *ATG16L*1 to the plasma membrane to initiate autophagosome formation and facilitate bacterial clearance. Disease-associated *NOD2* variants impair this recruitment, resulting in defective autophagy and reduced bacterial elimination (Lees et al., 2011; Li and Law, 2022). *ATG16L1* deficiency is also associated with increased reactive oxygen species production, impaired mitochondrial quality control, and enhanced inflammatory macrophage responses (Zhang et al., 2017b). In immune cells, the T300A variant has been shown to exert a dominant-negative effect, impairing autophagic induction and increasing inflammasome activation, including elevated IL-1β production (Gao et al., 2017). A key consequence of defective autophagy is the accumulation of p62/SQSTM1, which enhances NF-κB signaling and amplifies inflammatory pathways (Matsuzawa-Ishimoto et al., 2017).

Collectively, *ATG16L1* serves as a critical molecular link between microbial sensing, autophagosome formation, and intestinal homeostasis. The T300A variant disrupts multiple aspects of this pathway, including protein stability, WD40-mediated interactions, regulation of autophagic cargo, and inflammatory signaling, thereby contributing to increased susceptibility to intestinal inflammation.

### 3.3 IRGM

Immunity-related GTPase family M (*IRGM*) encodes an immunity-related GTPase that plays a pivotal role in autophagy initiation and intracellular pathogen clearance. IRGM promotes autophagy by interacting with *Beclin 1* and unc-51-like kinase 1 (*ULK1*), facilitating assembly of the autophagy initiation complex (Chauhan et al., 2015). Early studies demonstrated its protective role against *Mycobacterium tuberculosis* infection by activating antimicrobial autophagy (Budarf et al., 2009). Genetic analysis further revealed that deletions upstream of the *IRGM* risk allele reduce *IRGM* expression, thereby impairing antibacterial autophagic responses (Baxt and Xavier, 2015). Several *IRGM* polymorphisms, including rs13361189, rs10065172, and rs1000113, have been associated with increased susceptibility to CD (Glas et al., 2013). In addition to its role in canonical autophagy, *IRGM* interacts with *ATG16L1* and *NOD2* to coordinate autophagic responses to the intestinal microbiota (Chauhan et al., 2015). Importantly, *IRGM* also directly regulates mitophagy. It binds mitochondrial cardiolipin and translocates to mitochondria, where it modulates mitochondrial fission and induces Mitophagy to eliminate intracellular pathogens, including *Mycobacterium tuberculosis* (Singh et al., 2010). Furthermore, *ATG16L1*, peroxisomal biogenesis factor 13 (*PEX13*), and SMAD ubiquitination regulatory factor 1 (*SMURF1*) have been shown to regulate cytokine production and inflammasome activation through mitophagy-dependent mechanisms (Li and Law, 2022).

Collectively, genetic susceptibility loci associated with IBD form an interconnected network that governs mitochondrial quality control by integrating microbial sensing (e.g., *NOD2*), autophagosome formation (e.g., *ATG16L1* and *IRGM*), and organelle stress responses (e.g., X-box binding protein 1, XBP1), as summarized in Table 1. This coordinated genetic architecture provides a compelling rationale for the development of precision therapeutic strategies aimed at restoring mitophagy and mitochondrial homeostasis in IBD.

**TABLE 1:** Genetic variants associated with IBD and autophagy.

Gene	Autophagy-associated functions/Pathways	Association with IBD	References
*ATG16L1*	Forms a complex with ATG12-ATG5 and participates in autophagosome formation	The *ATG16L1* T300A (rs2241880) variant significantly increases CD risk; Affect Paneth cell secretion and dendritic cell antigen presentation	Saitoh et al. (2008), Wang et al. (2018), Hamade et al. (2024), Wei et al. (2025)
*IRGM*	Interacts with ULK1 and *Beclin 1* to promote autophagosome formation; Virus-induced autophagy	The risk allele rs10065172 in *IRGM* leads to reduced expression or impaired function, which compromises autophagy and intracellular bacterial clearance, thereby increasing the risk of CD	Budarf et al. (2009), Chauhan et al. (2015)
*NOD2*	Recognizes muramyl dipeptide (MDP) to activate autophagy, facilitating bacterial clearance Interacts with *ATG16L1* to promote autophagosome formation	*NOD2* is the gene with the highest genetic risk for CD; common mutations such as L1007fs lead to defective autophagy induction, impaired bacterial clearance capacity, and compromised intestinal barrier integrity	Negroni et al. (2018), Ashton et al. (2022)
*ULK1*	Targeted regulation of mTORC1 and AMPK	*ULK1* gene polymorphisms (rs12303764, rs3923716) are significantly associated with susceptibility to CD	Henckaerts et al. (2011), Kim et al. (2011)
*LRRK2*	Targeting *Beclin 1*, which is involved in autophagosome-lysosomal degradation	*LRRK2* polymorphisms (G2019S/N2081D) increase susceptibility to CD; upregulated expression associated with Paneth cell defects	Sun et al. (2024)
*XBP1*	Components of endoplasmic reticulum stress; Promotes autophagy via the XBP1/AMPK/mTOR pathway	The strongest associated variant in IBD is rs35873774; Reduced *XBP1* expression induces Paneth cell apoptosis, impairing cellular clearance capacity	Kaser et al. (2008), Meng et al. (2025)
*XIAP*	Regulation of autophagy via the NOD2-RIPK2-XIAP complex	The c.266delA mutation in the *XIAP* gene causes a severe reduction in *XIAP* expression, leading to enhanced apoptosis and impaired NOD2 signalling	Krieg et al. (2009), Parackova et al. (2020)
*VDR*	Induction of autophagy-related genes (such as *ATG16L1*) and regulation of autophagy via the miR-142 3p/ATG16L1 axis	Polymorphisms associated with increased IBD susceptibility; deficiency reduces autophagy and dysregulates Paneth cells	Sun, 2015; Wu et al. (2015), McGillis et al. (2021)
*MTMR3*	Negatively regulates autophagy; upregulation of its expression inhibits PRR-induced autophagy	In individuals with IBD who carry the rs713875 risk allele, elevated *MTMR3* expression leads to reduced autophagy and increased inflammatory cytokine production	Lahiri et al. (2015)
*SMURF1*	Virus autophagy; Participates in mitophagy via the C2 domain	A mononucleotide polymorphism in the *SMURF1* gene is associated with UC	Franke et al. (2010), Orvedahl et al. (2011)

## 4 The intestinal barrier and IBD

The intestinal barrier relies on two core components: the physical barrier and the chemical barrier. Because epithelial barrier dysfunction is a hallmark of both CD and UC, studies examining intestinal epithelial cells, goblet cells, and tight junction integrity provide mechanistic insights relevant to the broader spectrum of IBD.The physical barrier is primarily constituted by intestinal epithelial cells (IECs) and their intercellular junctional structures. IECs form a continuous cellular layer through specialized structures, including tight junctions (TJs), desmosomes, and adherens junctions (AJs), thereby regulating paracellular permeability (Neurath et al., 2025).

Among these, tight junctions are the most critical structures, formed by multiple transmembrane proteins including occludins, claudins, zonula occludens-1 (ZO-1), and junctional adhesion molecules (JAMs) (Huo et al., 2022; Mauduit et al., 2025). IECs are organized into crypts and villi (the small intestine possesses both, while the colon has only crypts), which together form the primary defense against external substances.

**FIGURE 2:**
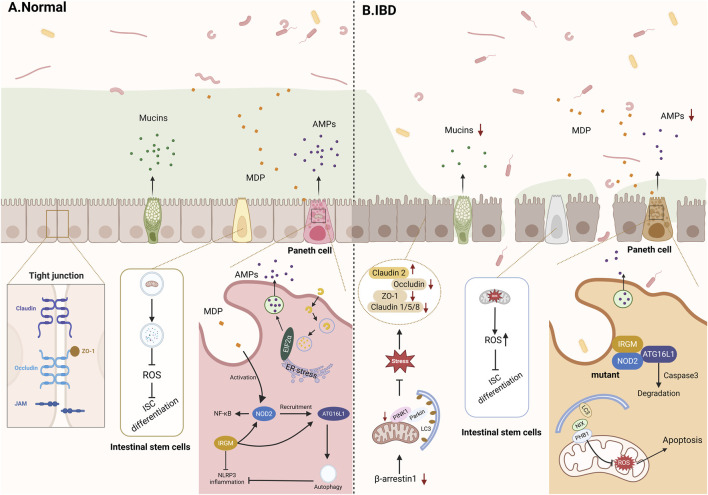
The role of mitochondria in maintaining intestinal barrier homeostasis. **(A)** Mitophagy maintains homeostasis in healthy epithelial cells. **(B)** In IBD, mitophagy interacts with intestinal tight junctions, intestinal stem cells, and dysfunctional Paneth cells, influencing barrier integrity. ZO-1, Zonula occludens-1; JAMs, Junctional adhesion molecule; ROS, Reactive oxygen species; ISC, Intestinal stem cells; MDP, Muramyl dipeptide; AMPs, Antimicrobial peptides; EIF2α, Eukaryotic initiation factor 2α; ER, Endoplasmic reticulum; NOD2, Nucleotide-binding oligomerization domain 2; ATG16L1, Autophagy related 16 like 1; IRGM, Immune-related GTPase family M; NF-κB, Nuclear factor kappa-B; PINK1, PTEN-induced kinase 1; Parkin, Parkin RBR E3 ubiquitin protein ligase; LC3, Microtubule-associated protein 1 light chain 3; Caspase3, Cysteine-dependent aspartate-specific protease 3; NIX, BCL2/adenovirus E1B 19-kDa-interacting protein 3-like; PHB1, Prohibitin 1.

The intestinal epithelium undergoes rapid and continuous renewal driven by leucine-rich repeat-containing G-protein-coupled receptor 5 (LGR5)+ intestinal stem cells (ISCs) located at the base of the crypts, with a typical turnover cycle of approximately 4–5 days (Barker et al., 2007). These stem cells differentiate into multiple specialized epithelial cell lineages, including enterocytes, goblet cells, Paneth cells, tuft cells, and enteroendocrine cells. Enterocytes represent the most abundant epithelial cell type. They are primarily responsible for nutrient absorption while also contributing to barrier integrity by forming tight junctions and regulating epithelial permeability. Goblet cells secrete highly glycosylated mucins that form a protective mucus layer covering the epithelial surface, thereby limiting microbial adhesion and invasion. Paneth cells, which are predominantly located in the crypts of the small intestine, play a crucial role in the chemical barrier by producing antimicrobial peptides, such as defensins, lysozyme, and regenerating islet-derived proteins, which suppress pathogenic microorganisms (Peterson and Artis, 2014). Tuft cells and enteroendocrine cells further contribute to epithelial defense by sensing luminal stimuli and regulating immune and neuroendocrine signaling pathways. Disruption of epithelial barrier integrity is a key pathogenic feature of IBD. Damage to epithelial cells, loss of tight junction integrity, and altered mucus production increase intestinal permeability, allowing luminal antigens and microorganisms to penetrate the mucosa and trigger excessive immune responses. In particular, dysfunction of Paneth cells has been associated with impaired antimicrobial peptide secretion and microbial dysbiosis, both of which contribute to chronic intestinal inflammation (Miao et al., 2025) (Figure 2).

Given the high metabolic demand and constant exposure of IECs to environmental stressors, mitochondrial function is pivotal for maintaining epithelial homeostasis. Emerging evidence suggests that mitochondrial quality control mechanisms, particularly mitophagy, are essential for preserving epithelial integrity and regulating inflammatory responses. Dysregulation of mitophagy can lead to mitochondrial damage, excessive ROS production, and epithelial barrier dysfunction, thereby contributing to the development and progression of IBD. Notably, accumulating evidence suggests that autophagy, beyond its role in mitochondrial quality control, is a fundamental regulator of intestinal homeostasis through its interactions with the gut microbiota and immune system. Disruption of autophagic processes has been shown to exacerbate intestinal inflammation, impair epithelial barrier integrity, and alter microbial composition, thereby contributing to IBD pathogenesis (Subramanian et al., 2024a; Subramanian et al., 2024b).

### 4.1 Mitophagy regulates intestinal tight junctions

Patients with IBD often exhibit an imbalance in the expression of TJ proteins. Clinical studies have shown that in CD patients, the expression of occludin, claudin-5, and claudin-8 is downregulated, while claudin-2 expression is upregulated, leading to altered tight junction structure and significant barrier dysfunction (Zeissig et al., 2007). Compared to CD patients, UC patients demonstrate even higher levels of claudin-2 expression (Zeissig et al., 2007). The dynamic maintenance of TJ structures is highly dependent on ATP supply, which is primarily derived from mitochondrial oxidative phosphorylation (OXPHOS). In Caco-2 cell models, ATP depletion disrupts the localization of claudin-7 and increases intestinal permeability, indicating that mitochondrial ATP production is essential for epithelial barrier integrity (JanssenDuijghuijsen et al., 2017). As the maintenance of intestinal epithelial junctions is energy-dependent, mitochondrial function may be central to maintaining epithelial barrier function. Wu et al. found that β-arrestin1 promotes the expression of intestinal TJ proteins claudin-1 and occludin by activating PINK1/Parkin-mediated mitophagy, thereby preserving intestinal barrier function; conversely, β-arrestin1 deficiency leads to reduced ATP production and mitochondrial dysfunction (Wu S. et al., 2024). Consistently, in IBD, upregulation of the TJ-associated molecule claudin-2 has been observed, and inactivation of claudin-2 has been shown to limit the progression of colitis in mice, suggesting claudin-2 as a potential therapeutic target for IBD (Raju et al., 2020). Ganapathy et al. demonstrated that LC3-mediated autophagy can enhance the TJ barrier by degrading claudin-2 (Ganapathy et al., 2022).

Additionally, studies have found that exposure to deoxynivalenol (DON) impairs cellular barrier function by altering the expression of TJ proteins. A strong negative correlation was observed between tight junction-associated proteins (ZO-1, claudin-3, occludin, and claudin-1) and mitophagy-related proteins (Parkin, PINK1, and LC3II/I), suggesting that TJ disruption is associated with dysregulated mitophagy (Zhang C. et al., 2022). Impaired mitophagy also leads to the accumulation of reactive oxygen species (ROS), triggering phosphorylation of zonula occludens-1 (ZO-1) and thereby compromising the integrity of TJ proteins (Festa et al., 2018). In summary, mitophagy maintains the integrity and function of intestinal tight junctions through multiple pathways, including energy supply and specific protein degradation, and its deficiency is closely linked to the pathogenesis of IBD.

### 4.2 Mitophagy regulates intestinal epithelial cells

#### 4.2.1 Goblet cells

Colonic mucus is primarily produced by goblet cells. It consists of two layers: an outer layer composed of secreted gel-forming mucins (MUC2, MUC5AC, MUC5B, and MUC6), which constitute the major components of the mucus layer and provide its viscoelastic properties, and an inner layer formed by transmembrane mucins (MUC1, MUC3A/B, MUC4, MUC12, MUC17) that create a dense glycocalyx, acting as a protective barrier between the secreted mucins and the epithelial cells (Byrd and Bresalier, 2004; Tailford et al., 2015). In the colon, the outer mucus layer facilitates bacterial colonization, while the inner layer possesses antimicrobial properties (Wang and Shen, 2024). Consistent with this protective role, analysis of rectal biopsy samples from IBD patients revealed that both mucus layer thickness and goblet cell density were significantly lower in active-disease patients compared to normal controls (Strugala et al., 2008). van der Post et al. observed a marked reduction in the expression of the main structural component mucin 2 (MUC2) in active UC patients, accompanied by abnormal bacterial penetration into the inner mucus layer (van der Post et al., 2019). Similarly, Dorofeyev et al. reported aberrant expression of MUC2, MUC3, and MUC4 genes in CD patients (Dorofeyev et al., 2013). Furthermore, comparisons between UC and CD indicate that the mucus layer in UC patients is thinner and more discontinuous, with MUC2 expression further decreased during active UC. This reduction in MUC2 secretion may result from abnormalities in translation or post-translational modifications, as well as reduced goblet cell numbers (Kang et al., 2022). Collectively, this evidence consistently indicates that alterations in mucin expression and function play a distinct role in IBD. Emerging evidence suggests that autophagy-related processes are closely involved in mucin regulation. Mitophagy is closely associated with the synthesis and secretion of MUC2 mucin. Research has shown that viral infection can upregulate NOD-like receptor X1 (NLRX1), thereby activating mitophagy and subsequently suppressing MUC2 expression via the extracellular signal-regulated kinase (ERK)/myosin light chain kinase (MLCK) signaling pathway, leading to disruption of the intestinal mucosal barrier (Tao et al., 2024). Additionally, activation of autophagy upregulates MUC5AC expression through the JNK-AP-1 pathway, and autophagy inhibitors such as bafilomycin A1, or knockdown of key autophagy genes (*Beclin 1*, *ATG5*) significantly inhibit MUC5AC expression, indicating that autophagy regulates MUC5AC (Ye et al., 2019). However, whether mitophagy exerts specific regulatory effects on MUC proteins remains to be fully elucidated. Although accumulating evidence links autophagy to mucin synthesis and secretion, the specific contribution of mitophagy to goblet cell function remains poorly defined, representing an essential gap in our understanding of epithelial barrier regulation in IBD.

#### 4.2.2 Paneth cells

Paneth cells are secretory cells located at the base of the small intestinal crypts. They contribute to host defense against pathogens and the maintenance of intestinal homeostasis by secreting antimicrobial peptides (AMPs), such as human defensin-5 (HD5) and human defensin-6 (HD6), as well as lysozyme (Salzman et al., 2010; Bevins and Salzman, 2011; Clevers and Bevins, 2013). In addition to AMPs, Paneth cells also release a range of inflammatory cytokines, including tumor necrosis factor-α (TNF-α), transforming growth factor-β1 (TGF-β1), and prostaglandin E2, as well as growth factors such as epidermal growth factor (EGF). Through these secretory products, Paneth cells support intestinal stem cell proliferation and maintenance, primarily by regulating Notch and Wnt signaling pathways, which are essential for epithelial renewal and tissue repair (Sato et al., 2011; Clevers and Bevins, 2013). In CD patients, the expression of α-defensins in Paneth cells is significantly downregulated, weakening mucosal antibacterial defense and leading to intestinal dysbiosis (Wehkamp et al., 2005). Increasing evidence suggests that autophagy plays a crucial role in Paneth cell secretory function. During infection with invasive pathogens, disruption of the endoplasmic reticulum (ER)-Golgi apparatus can impair conventional lysozyme secretion. Under these conditions, ER stress activates an alternative secretion mechanism known as secretory autophagy, which is mediated through the protein kinase RNA-like endoplasmic reticulum kinase (PERK)-eukaryotic initiation factor 2α (eIF2α) signaling pathway. This pathway enables the directional transport and release of lysozyme through autophagy-related vesicles, thereby enhancing host defense against bacterial invasion (Bel et al., 2017).

Mitochondrial dysfunction has also been implicated in Paneth cell abnormalities. Jackson et al. demonstrated that loss of Prohibitin-1 (PHB1) induces mitochondrial dysfunction and leads to Paneth cell defects characterized by impaired antimicrobial peptide expression and intestinal inflammation. Treatment with the mitochondria-targeted antioxidant Mito-Tempo restored antimicrobial peptide expression and ameliorated ileitis in PHB1-deficient mice, suggesting that excessive mitochondrial reactive oxygen species (mtROS) contribute to Paneth cell dysfunction (Jackson et al., 2020). Furthermore, increased mtROS levels can promote the interaction between PHB1 and NIX (also known as BNIP3L) with microtubule-associated protein 1 light chain 3 (LC3), a key autophagosomal membrane protein involved in autophagy. The binding of NIX to LC3-II facilitates mitophagy activation and reduces apoptosis, thereby linking mitochondrial stress to mitophagy-mediated regulation of Paneth cell survival (Alula et al., 2023).

Mutations in IBD-associated autophagy genes (*ATG16L1*, *NOD2*, and *IRGM*) can directly impair the autophagic function of Paneth cells. Mice carrying the *ATG16L1* T300A mutation exhibit reduced antibacterial autophagy and abnormal lysozyme distribution in Paneth cells (Bel et al., 2017). The T300A variant is more susceptible to cleavage by caspase-3, leading to decreased ATG16L1 protein levels and reduced autophagic flux (Lassen et al., 2014; Murthy et al., 2014). *NOD2* regulates the expression and secretion of antimicrobial peptides; its mutation impairs the NF-κB and autophagic responses to intracellular bacteria, leading to bacterial accumulation within intestinal epithelial cells (Sidiq et al., 2016). *NOD2* mutation also reduces the recruitment of *ATG16L1*, which is another mechanism for impaired autophagy-mediated clearance of intracellular bacteria (Baxt and Xavier, 2015). Additionally, *Irgm1*-deficient mice exhibit alterations in Paneth cell location and granule morphology, reduced AMP expression, and consequent intestinal injury. Mitophagy is impaired in the Paneth cells of *Irgm1* knockout mice, indicating that *Irgm1* regulates mitophagy to ensure the proper processing of Paneth cell granules (Liu et al., 2013). These studies demonstrate that mitophagy is a key regulator of Paneth cell homeostasis and that mitophagy dysfunction can be associated with Paneth cell abnormalities. Together, these findings position mitophagy as a central regulator of Paneth cell antimicrobial function, linking genetic susceptibility, mitochondrial stress, and epithelial innate immunity in the pathogenesis of IBD.

## 5 Immune cells and mitophagy

The intestinal immune system comprises innate and adaptive immune responses. The innate immune response serves as the first line of defense against pathogens, mediated by various cell types, including epithelial cells, macrophages, dendritic cells (DCs), monocytes, neutrophils, and natural killer cells (Zhang and Li, 2014; Bergman et al., 2020). It triggers inflammation by producing cytokines, chemokines, and antimicrobial peptides, leading to phagocytosis, antigen presentation, and activation of the adaptive immune system (Bergman et al., 2020). These cells recognize microbial antigens via pattern recognition receptors and participate in mechanisms such as autophagy to maintain cellular homeostasis (Zhang and Li, 2014).

**FIGURE 3:**
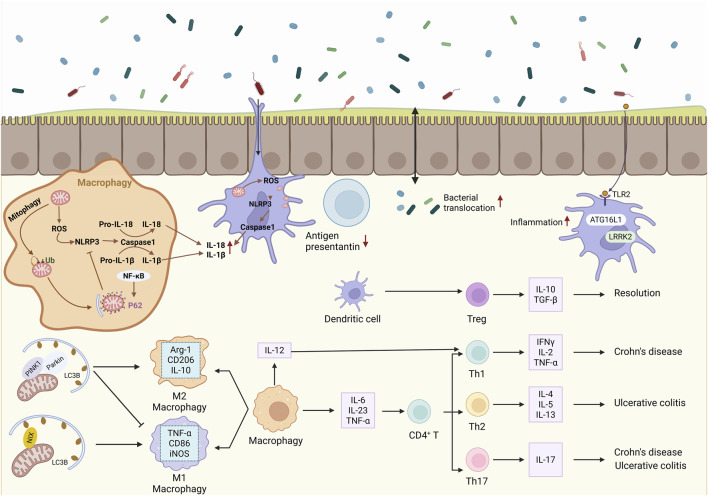
Regulation of immune cells in IBD through mitophagy. Schematic illustrating mitophagy-mediated regulation of intestinal immune responses in IBD. Mitophagy modulates macrophage polarization between M1 and M2 phenotypes, regulates NLRP3 inflammasome activation, affects dendritic cell antigen processing and presentation, and shapes T cell differentiation toward effector or regulatory subsets, collectively contributing to intestinal immune homeostasis. ROS, Reactive oxygen species; NLRP3, NOD-like receptor family pyrin domain containing 3; NF-κB, Nuclear factor kappa-B; Ub, Ubiquitin; p62, Sequestosome 1; PINK1, PTEN-induced kinase 1; Parkin, Parkin RBR E3 ubiquitin protein ligase; LC3, Microtubule-associated protein 1 light chain 3; NIX, BCL2/adenovirus E1B 19-kDa-interacting protein 3-like; TLR2, Toll-like receptor 2; ATG16L1, Autophagy related 16 like 1; LRRK2, Leucine-rich repeat kinase 2.

Accordingly, this section focuses on the role of mitophagy in regulating the function of key intestinal innate immune cells, particularly macrophages and dendritic cells, and its relevance to IBD pathogenesis (Figure 3). In addition, accumulating evidence indicates that mitophagy is equally critical for adaptive immune cell differentiation and immune tolerance, which is briefly discussed below.

### 5.1 Macrophages

Mitochondrial quality control is increasingly recognized as a central determinant of macrophage inflammatory status in the intestinal microenvironment. Intestinal macrophages primarily derive from monocytes in the bone marrow. Under physiological conditions, they help maintain intestinal epithelial cell numbers and tissue homeostasis by phagocytosing apoptotic epithelial cells (Cummings et al., 2016; Shaw et al., 2018). Macrophages can polarize from a resting state (M0) into pro-inflammatory macrophages (M1) or anti-inflammatory macrophages (M2) phenotypes. This polarization is central to the pathogenesis of IBD, as well as to the regulation of intestinal homeostasis and tissue repair (Lissner et al., 2015; Zhang et al., 2023; Lu et al., 2024).

#### 5.1.1 Mitophagy affects macrophage phenotypic changes

In IBD patients, the number of macrophages in the intestinal mucosa is significantly increased, accompanied by a phenotypic shift from the M2 to the M1 (Lissner et al., 2015). This shift is characterized by increased expression of M1-associated markers, including inducible nitric oxide synthase (iNOS), cluster of differentiation 86 (CD86), and tumor necrosis factor-α (TNF-α), whereas markers associated with the M2 phenotype, such as arginase-1 (Arg1), cluster of differentiation 206 (CD206), and interleukin-10 (IL-10), are significantly downregulated. These alterations promote excessive inflammatory responses and contribute to intestinal tissue damage (Lissner et al., 2015; Bain and Schridde, 2018; Moreira Lopes et al., 2020). Therefore, promoting macrophage differentiation into the M2 anti-inflammatory phenotype is considered a promising therapeutic strategy for IBD. Numerous studies have revealed that mitophagy plays an important role in regulating macrophage polarization. One of the most extensively studied pathways involves PTEN-induced putative kinase 1 (PINK1) and the E3 ubiquitin ligase Parkin, which together mediate mitochondrial quality control. Activation of the PINK1/Parkin signaling pathway promotes mitophagy and has been shown to suppress the expression of arsenic-induced M1-associated inflammatory mediators, including iNOS and TNF-α, while promoting macrophage polarization toward the M2 phenotype (Qu et al., 2022). In addition to PINK1/Parkin signaling, other mitophagy regulators also contribute to macrophage functional programming. NIX (BNIP3L), a mitophagy receptor located on the outer mitochondrial membrane, has been shown to influence macrophage inflammatory responses. Deficiency of NIX reduces the production of M1-associated pro-inflammatory cytokines, further supporting the regulatory role of mitophagy in macrophage polarization (Wang S. et al., 2023). Genetic variants affecting autophagy pathways can also disrupt macrophage mitochondrial homeostasis. For example, macrophages derived from individuals carrying the autophagy-related protein 16-like 1 *(*ATG16L1) T300A risk variant exhibit elevated ROS levels, impaired mitophagy, and reduced bacterial fusion with the lysosomal marker lysosome-associated membrane protein 1 (LAMP1). These defects ultimately result in impaired bacterial clearance and diminished major histocompatibility complex class II (MHC II) antigen processing (Zhang et al., 2017b).

Metabolic signaling pathways further connect mitophagy to macrophage polarization. The anti-inflammatory cytokine interleukin-10 (IL-10) has been shown to induce mitophagy by inhibiting the activity of the mechanistic target of rapamycin (mTOR) pathway. This process delays glycolytic activation following lipopolysaccharide (LPS) stimulation and promotes macrophage polarization toward the anti-inflammatory M2 phenotype (Wang S. et al., 2023). Consistently, pharmacological activation of mitophagy using rapamycin, an mTOR inhibitor, suppresses M1 macrophage polarization and facilitates the transition toward the M2 phenotype (Wang S. et al., 2023; Guo et al., 2025). In contrast, inhibition of mitophagy using 3-methyladenine (3-MA) promotes macrophage polarization toward the pro-inflammatory M1 phenotype (Guo et al., 2025). Together, these findings indicate that mitophagy functions as a regulatory checkpoint that integrates mitochondrial stress signals with macrophage polarization programs. However, the molecular mechanisms by which mitophagy fine-tunes macrophage phenotypic plasticity remain incompletely understood.

#### 5.1.2 Mitophagy regulates macrophage metabolic reprogramming

As macrophage polarization is strongly dependent on metabolic status, mitophagy-driven mitochondrial quality control is critical for regulating metabolic reprogramming. By selectively removing damaged mitochondria, mitophagy maintains mitochondrial integrity and preserves the metabolic flexibility required for macrophage phenotypic transformation. Accumulating evidence indicates that pro-inflammatory M1 macrophages primarily rely on aerobic glycolysis, which is characterized by enhanced glycolytic flux and disruption of the tricarboxylic acid (TCA) cycle, whereas anti-inflammatory M2 macrophages depend predominantly on mitochondrial oxidative phosphorylation (OXPHOS) to meet their energy demands (Zhang et al., 2023). During mitochondrial dysfunction, damaged mitochondria generate excessive ROS and accumulate TCA cycle intermediates such as succinate. Elevated succinate levels can stabilize hypoxia-inducible factor 1α (HIF-1α), a transcription factor that promotes glycolytic metabolism and the expression of pro-inflammatory genes. This metabolic shift toward glycolysis reinforces the inflammatory phenotype of M1 macrophages and amplifies inflammatory responses (Kelly and O’Neill, 2015). At the same time, mitochondrial damage and energy imbalance can suppress the activity of AMP-activated protein kinase (AMPK). This key metabolic sensor promotes mitochondrial biogenesis and supports oxidative metabolism. Inhibition of AMPK limits macrophages’ ability to transition to oxidative phosphorylation, thereby maintaining them in a glycolysis-dominant, pro-inflammatory state (Kelly and O’Neill, 2015). Mitophagy plays a crucial role in preventing these metabolic disturbances by eliminating dysfunctional mitochondria and preserving mitochondrial respiratory capacity. Through this process, mitophagy supports efficient oxidative phosphorylation and promotes metabolic conditions favorable for M2 macrophage polarization. Recent studies have shown that spermidine, a naturally occurring polyamine, can activate AMPK signaling, enhance autophagy and mitophagy, and modulate metabolic pathways in macrophages. Activation of this pathway has been shown to alleviate DSS-induced colitis in mouse models, highlighting the protective role of mitophagy-mediated metabolic regulation in intestinal inflammation (Liu et al., 2020). In summary, these findings suggest that mitophagy serves as a critical regulator linking mitochondrial quality control with macrophage metabolic reprogramming. By maintaining mitochondrial function and supporting oxidative metabolism, mitophagy stabilizes of the anti-inflammatory M2 macrophage phenotype and helps limit excessive intestinal inflammation in IBD.

#### 5.1.3 Mitophagy regulates the NLRP3 inflammasome

Mitophagy is also crucial for regulating the NLR family pyrin domain-containing 3 (NLRP3) inflammasome within macrophages. The NLRP3 inflammasome is a cytoplasmic multiprotein complex consisting of the sensor protein NLRP3, the adaptor protein apoptosis-associated speck-like protein containing a caspase recruitment domain, and the effector protein caspase-1 (Zhen and Zhang, 2019). It recognizes damage-associated molecular patterns (DAMPs) or pathogen-associated molecular patterns (PAMPs) to sense microbial or endogenous danger signals. NLRP3 inflammasome activation requires an initial priming signal mediated by NF-κB activation, followed by an activation step driven by microbial or danger-associated signals (Zhen and Zhang, 2019; Zheng et al., 2020). Activation of atypical inflammasomes involves caspase-11/4/5-mediated cleavage of gasdermin-D (GSDMD), leading to pyroptosis or activation of the NLRP3 inflammasome complex (Zheng et al., 2020). In the intestinal mucosa of IBD patients, the expression and activation levels of the NLRP3 inflammasome and its downstream signaling molecules (Caspase-1, IL-1β, IL-18) are significantly upregulated (Liu et al., 2017). Emerging evidence indicates that mitophagy plays an important role in limiting NLRP3 inflammasome activation by maintaining mitochondrial quality and reducing inflammatory signaling. For instance, NF-κB activation can induce the accumulation of the autophagy receptor sequestosome 1 (p62/SQSTM1), which translocates to damaged mitochondria and promotes their removal via mitophagy. This process reduces mitochondrial ROS and prevents excessive activation of the NLRP3 inflammasome (Zhong et al., 2016). Autophagy-related mechanisms also contribute to the regulation of inflammasomes. Autophagosomes can suppress NLRP3 inflammasome activation by limiting mitochondrial ROS production and removing damaged mitochondria. Conversely, deficiency of key autophagy components such as LC3B and *Beclin-1* leads to enhanced caspase-1 activation and increased secretion of IL-1β and IL-18, thereby amplifying inflammatory responses (Nakahira et al., 2011). In addition to indirectly regulating inflammasome activation through mitochondrial quality control, mitophagy can also directly target inflammasome components for degradation. Ubiquitination of assembled inflammasome complexes promotes recruitment of p62, which facilitates their sequestration into autophagosomes. The observed colocalization of ASC with autophagosomal structures suggests that NLRP3 inflammasome complexes can be selectively degraded through autophagy-dependent pathways (Shi et al., 2012).

Furthermore, the IBD-associated autophagy gene *IRGM* limits inflammasome activity by activating autophagy to induce degradation of NLRP3 and ASC, and it suppresses pyroptosis and intestinal inflammation in experimental colitis models (Mehto et al., 2019). Collectively, these studies demonstrate that mitophagy serves as a critical negative regulator of NLRP3 inflammasome activation, thereby limiting excessive inflammatory responses in IBD.

### 5.2 Dendritic cells

In addition to macrophages, mitophagy critically shapes dendritic cell survival, activation thresholds, and antigen-presenting capacity in the intestinal mucosa. The primary function of intestinal dendritic cells (DCs) is to capture, process, and present antigens, thereby initiating immune responses. Under physiological conditions, most DCs exist in an immature state, characterized by low surface expression of major histocompatibility complex class II (MHC II) and co-stimulatory molecules, while retaining the potential to induce T cell differentiation (Iwasaki and Medzhitov, 2015; Sun et al., 2022). DC-derived thymic stromal lymphopoietin (TSLP) restrains Th17 cell differentiation and promotes regulatory T cell (Treg) development, thereby exerting a protective effect in colitis models (Spadoni et al., 2012). In IBD patients, intestinal mucosal DCs are significantly elevated and exhibit a strongly activated state, characterized by increased surface expression of MHC-II, co-stimulatory molecules (CD80, CD86), and Toll-like receptors (TLR2, TLR4), along with heightened production of pro-inflammatory cytokines such as TNF-α and IL-8 (Bell et al., 2001; Hart et al., 2005; Baumgart et al., 2009). Together, these changes indicate enhanced recruitment and activation of DCs during acute disease phases, thereby promoting intestinal inflammation.

Viral infection suppresses autophagy via the AKT/mTOR pathway, thereby promoting the expression of CD83 and pro-inflammatory cytokines (IL-6, IL-12, TNF-α) in DCs (Liu et al., 2024b). Conversely, PINK1/Parkin pathway-mediated mitophagy clears damaged mitochondria in DCs and curbs excessive apoptosis, thus modulating immune function (Zhang Y. et al., 2022). Activation of general control nonderepressible 2 (GCN2) in DCs enhances mitophagy to remove damaged mitochondria, thereby suppressing ROS-dependent NLRP3 inflammasome activation, reducing IL-1β production, and subsequently limiting Th17 responses and alleviating inflammation in mice (Ravindran et al., 2016). Consistent with these findings, defects or dysregulation of autophagy-related genes impair DC function and promote intestinal inflammation. Knockout of autophagy genes such as *ATG16L1*, *ATG7*, and *Beclin 1* significantly compromises DC activation, antigen presentation, and the capacity to mediate T-cell differentiation (Reed et al., 2013; Bhattacharya et al., 2014; Zhang et al., 2017a). Further studies demonstrate that DCs from CD patients carrying the *ATG16L1* T300A allele exhibit reduced bacterial particle uptake, dysregulation of immune activation markers (HLA-DR, CD86), and diminished trans-epithelial dendrite formation, resulting in impaired antigen capture (Strisciuglio et al., 2013). In CD patients, elevated leucine-rich repeat kinase 2 (LRRK2) expression in DCs inactivates *Beclin 1* and suppresses autophagy, augmenting Dectin-1-driven NF-κB activation and pro-inflammatory cytokine production, thereby worsening intestinal inflammation in experimental models (Takagawa et al., 2018). These findings highlight that intact mitophagy is essential for maintaining dendritic cell functional homeostasis and preventing exaggerated inflammatory signaling. Moreover, mitophagy participates in antigen presentation and promotes adaptive immune responses. When DCs form immune synapses with T cells, mitochondria accumulate at the synaptic region and undergo partial depolarization, triggering mitophagy to protect DCs from damage during antigen presentation and T cell activation (Gómez-Cabañas et al., 2019). Overall, mitophagy preserves intestinal immune homeostasis by regulating mitochondrial quality, antigen presentation, and inflammatory signaling in dendritic cells during IBD.

### 5.3 Adaptive immune cells

Adaptive immune responses in the intestine are mediated by T and B lymphocytes, whose activation, differentiation, and effector functions are closely associated with mitochondrial metabolism and quality control. Among T cells, naive T cells (Th0) differentiate into various effector subsets following antigen stimulation. Specifically, Th1 cells, induced by IL-12, primarily secrete IFN-γ, TNF-α, and IL-2, whereas Th2 cells produce cytokines including IL-4, IL-5, and IL-13 (Romagnani, 1994). In IBD, CD is generally considered to be driven by both Th1 and Th17 responses; in contrast, UC appears to be predominantly mediated by a Th2-type response (Monteleone et al., 1997; Geremia et al., 2014). Studies have shown that mucosal T cells from CD patients secrete higher levels of IL-2 and IFN-γ (Breese et al., 1993; Noguchi et al., 1995); by comparison, those from UC patients produce more IL-5 and IL-13 (Fuss et al., 2004; Heller et al., 2005). Furthermore, Th17 cells promote persistent intestinal inflammation by secreting pro-inflammatory cytokines such as IL-17 (Hu et al., 2025). By contrast, regulatory T (Treg) cells help maintain immune homeostasis by producing anti-inflammatory cytokines, including IL-10 and TGF-β (Hu et al., 2025).

Recent studies have highlighted the critical role of mitophagy in T cell development and differentiation. For example, an imbalance in the Th17/Treg ratio can disrupt intestinal immune homeostasis and induce colitis in mice (Britton et al., 2019). Research reveals that a lack of autophagy-related gene *Atg7* leads to increased Th1 cells and reduced Treg cells, thereby exacerbating intestinal inflammation in mice (Zhou et al., 2025). Similarly, deletion of *ATG16L1* in T cells elevates Th2 responses while decreasing the number of Foxp3+ Treg cells (Kabat et al., 2016). Moreover, mitophagy is reduced during Th1 differentiation induction. Notably, PINK1 deficiency promotes Th1 differentiation and increases the number of Th1 cells in colitis models (Mei et al., 2023). In addition, the natural compound Bergapten enhances mitophagy via the PPARγ/NF-κB signaling pathway, thereby modulating the Th17/Treg balance and ameliorating colitis (Xu et al., 2024). Disruption of mitophagy, therefore, skews T-cell lineage commitment and undermines immune tolerance in the intestinal environment. Together, these findings indicate that mitophagy is a key regulator of T-cell differentiation and immune balance, with direct implications for IBD pathogenesis.

## 6 Mitophagy in regulating IBD pathogenesis

**FIGURE 4:**
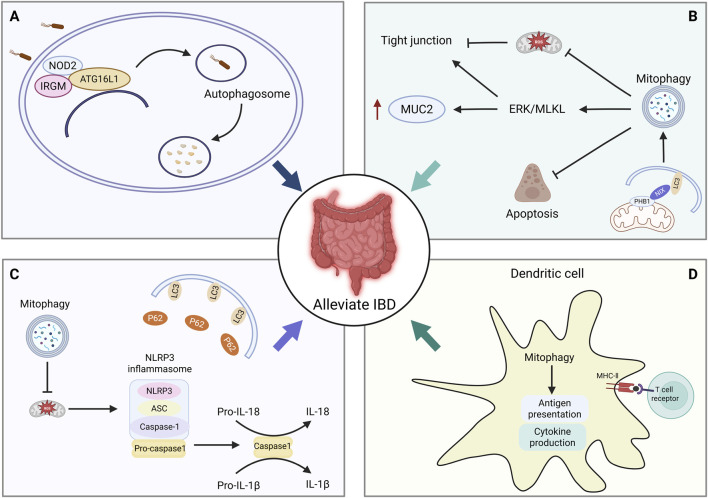
Mechanisms by which mitophagy alleviates IBD. **(A)** Autophagy-related gene variants in IBD. **(B)** Mitophagy regulates intestinal tight junctions, mucin secretion, and Paneth cell apoptosis. **(C)** Mitophagy regulates NLRP3 inflammasome activation. **(D)** Mitophagy regulates antigen presentation, inflammatory cytokine secretion in dendritic cells, and T cell differentiation. IBD, Inflammatory Bowel Disease; NOD2, Nucleotide-binding oligomerization domain 2; ATG16L1, Autophagy related 16 like 1; IRGM, Immune-related GTPase family M; ROS, Reactive oxygen species; LC3, Microtubule-associated protein 1 light chain 3; Caspase3, Cysteine-dependent aspartate-specific protease 3; NIX, BCL2/adenovirus E1B 19-kDa-interacting protein 3-like; PHB1, Prohibitin 1; MUC2, Mucin 2; ERK, Extracellular regulated protein kinases; MLKL, Mixed Lineage Kinase Domain-Like; NLRP3, NOD-like receptor family pyrin domain containing 3; MHC-II,Major histocompatibility complex class II.

The pathogenesis of IBD is closely associated with dysregulated mitophagy. In a broader context, autophagy serves as a critical interface linking host immunity, intestinal microbiota, and metabolic homeostasis, all of which are central to IBD development. Recent studies have highlighted that impaired autophagic flux not only disrupts mitochondrial quality control but also promotes microbial dysbiosis, aberrant immune activation, and sustained intestinal inflammation, underscoring its importance as a key pathogenic mechanism and a potential therapeutic target in IBD (Subramanian et al., 2024a; Subramanian et al., 2024b). Mitophagy is the selective clearance of dysfunctional mitochondria, primarily through the PINK1–Parkin pathway and receptor-mediated pathways (BNIP3, NIX, FUNDC1), to maintain mitochondrial quality, reduce reactive oxygen species (ROS) accumulation, and suppress inflammatory signaling (Figure 4).

Genome-wide association studies have identified variants in multiple autophagy-related genes (e.g., *ATG16L1*, *NOD2*, *IRGM*) as significant risk factors for IBD, particularly CD. These variants impair xenophagy and mitophagy, leading to defective intracellular bacterial clearance, Paneth cell dysfunction, and compromised epithelial barrier integrity, thereby forming the genetic basis of IBD (Miao et al., 2025). Intestinal epithelial barrier integrity is highly dependent on mitochondrial energy production. Mitophagy maintains efficient oxidative phosphorylation to support the dynamic balance and function of tight junction proteins by timely removing damaged mitochondria. Impaired mitophagy leads to ROS accumulation, which causes phosphorylation of ZO-1 and upregulation of claudin-2, thereby increasing epithelial permeability (Festa et al., 2018; Ganapathy et al., 2022). It also compromises antimicrobial peptide secretion by Paneth cells, weakening the chemical barrier. In innate immunity, mitophagy regulates metabolic reprogramming and phenotypic polarization of macrophages. Effective mitophagy supports OXPHOS and M2 macrophage polarization. Its deficiency results in damaged mitochondrial dysfunction, which stabilizes HIF-1α via succinate and ROS, drives glycolytic metabolism, sustains the M1 state, and hyperactivates the NLRP3 inflammasome, releasing excessive pro-inflammatory cytokines (Kelly and O’Neill, 2015). In dendritic cells, mitophagy modulates antigen presentation and inflammatory signaling (Gómez-Cabañas et al., 2019). Regarding adaptive immunity, mitophagy influences the balance of T cell differentiation; its dysfunction can disrupt the Th17/Treg equilibrium, exacerbating colitis development (Britton et al., 2019).

In summary, mitophagy performs a crucial quality-control function in IBD through a coordinated network involving epithelial cells, Paneth cells, and various immune cells. Targeting mitophagy-related pathways may therefore represent a promising strategy to selectively modulate epithelial integrity and immune metabolism in IBD.

## 7 Therapeutic compounds targeting mitophagy

The development of mitophagy-specific modulators for IBD is still in its early stages. Yet, several compounds have shown potential to regulate this process. Therapeutic agents targeting mitophagy in IBD can be broadly classified into three categories: natural compounds, synthetic compounds, and probiotics. Most of these compounds act on the PINK1-Parkin-mediated pathway, while others modulate BNIP3L-, BNIP3-, FUNDC1-, and LC3-mediated pathways. Transcriptional regulators, such as Peroxisome Proliferator-Activated Receptor (PPAR) and its coactivator PGC-1α, also promote mitophagy (Wu H. et al., 2024). Compounds like metformin and berberine confer protective effects in IBD models, partly by activating AMPK or promoting mitochondrial biogenesis (Guo et al., 2023; Um et al., 2023).

Importantly, several natural compounds reported to alleviate intestinal inflammation have also been shown to influence mitophagy. For example, curcumin has been reported to activate the AMPK signaling pathway and enhance PINK1/Parkin-mediated mitophagy, thereby reducing mitochondrial ROS accumulation and inflammatory cytokine production (Yu et al., 2026). Similarly, resveratrol promotes mitochondrial quality control through the SIRT1-AMPK axis, thereby enhancing mitophagy and improving mitochondrial function in inflammatory conditions (Liu H. et al., 2025). In addition, certain probiotics, including *Lactobacillus* and *Bifidobacterium* species, may indirectly regulate mitophagy by reducing oxidative stress, modulating host autophagy-related gene expression, and restoring intestinal microbial balance (Wang R. et al., 2023; Liu X. et al., 2025). These effects can contribute to improved epithelial barrier integrity and attenuation of intestinal inflammation.

However, it should be noted that many of these interventions exert pleiotropic biological effects, simultaneously influencing oxidative stress responses, immune signaling pathways, and gut microbiota composition. For instance, curcumin has been reported to promote Parkin-dependent mitophagy via the AMPK-TFEB signaling pathway in intestinal epithelial cells, thereby improving mitochondrial quality control (Cao et al., 2020). At the same time, curcumin can suppress multiple inflammatory pathways, including NF-κB, ERK1/2, and JNK signaling, inhibit activation of the NLRP3 inflammasome, and directly scavenge ROS (Iglesias et al., 2022). In experimental colitis models, its protective effects have also been attributed to reductions in oxidative stress and restoration of mitochondrial function. Similarly, resveratrol activates the SIRT1-AMPK signaling axis, which enhances mitophagy and also regulates broader mitochondrial processes, including PGC-1α-mediated mitochondrial biogenesis, NAD^+^ metabolism, and NF-κB-driven inflammation (Price et al., 2012; Ku et al., 2021).

Conversely, probiotics may further illustrate this multi-target phenomenon. Certain strains, such as *Lactobacillus rhamnosus* and *Bacillus* species, have been shown to regulate intestinal homeostasis by simultaneously modulating autophagic flux, tight-junction integrity, inflammatory signaling pathways, and apoptosis, making it difficult to isolate the specific contribution of mitophagy (Wu et al., 2019; Zhao et al., 2023; Orlando et al., 2025). Consequently, the therapeutic benefits observed in IBD models cannot always be attributed solely to mitophagy modulation. Instead, mitophagy likely functions as one component of a broader regulatory network involving mitochondrial metabolism, immune signaling, and microbial interactions.

Moreover, relatively few studies have employed mitophagy-specific inhibitors or genetic models to directly determine whether activation of mitophagy is the primary driver of therapeutic outcomes. As a result, although many compounds have been shown to both activate mitophagy and reduce intestinal inflammation, the causal contribution of mitophagy to their protective effects remains incompletely defined. Further mechanistic studies are therefore required to clarify the extent to which mitophagy independently contributes to the therapeutic efficacy of these interventions in IBD. Representative compounds and their reported mechanisms of mitophagy modulation in IBD are summarized in Table 2. Overall, therapeutic strategies that regulate mitophagy represent a promising approach for IBD management and may complement existing anti-inflammatory treatments.

**TABLE 2:** Therapeutic compounds targeting mitophagy.

Names	Target/Signaling pathway	Molecular mechanism	References
Curcumin	PINK1/Parkin	Curcumin effectively ameliorates oxidative stress, intestinal epithelial barrier damage, and mitochondrial impairment by activating PINK1–Parkin-mediated mitophagy	Cao et al. (2020), Jin et al. (2022)
Resveratrol	PINK1/Parkin	Resveratrol can promote mitophagy via the PINK1/Parkin pathway and inhibit the activation of the NLRP3 inflammasome	Fan et al. (2021)
	BNIP3	Resveratrol upregulates BNIP3-related mitophagy via HIF1 and AMPK, thereby alleviating mitochondrial damage and oxidative stress	Li et al. (2020)
Spermidine	PINK1/Parkin	Spermidine activates the PINK1/Parkin pathway to induce mitophagy, which alleviates mitochondrial dysfunction caused by PINK1 depletion	Qi et al. (2016), Jang et al. (2023)
Quercetin	PPARγ/PGC-1α/NF-κB	Quercetin enhances mitophagy by targeting the PPARγ/PGC-1α/NF-κB axis, effectively inhibiting apoptosis and the accumulation of inflammatory factors	Wu et al. (2024a)
	PINK1/Parkin	Quercetin treatment promoted the expression levels of Parkin and PINK1, reduced the accumulation of mtROS, and decreased the activation of the NLRP3 inflammasome	Han et al. (2021)
	BNIP3	Quercetin improves intestinal barrier function by enhancing BNIP3-mediated mitophagy, ameliorating intestinal permeability, and activating the TGF-β signaling pathway	Xiao et al. (2026)
Bergamottin	LC3	Bergamottin promotes mitophagy to inhibit the activation of the NLRP3 inflammasome and pyroptosis	Luo et al. (2023)
	PPARγ/NF-κB	Bergamottin enhances mitophagy and activates PPARγ/NF-κB signaling, ameliorating intestinal barrier damage and Th17/Treg imbalance in a mouse model of colitis	Xu et al. (2024)
Myrtenol	ANXA1/PINK1/Parkin	Myrtenol enhances mitophagy via the ANXA1/PINK1/Parkin pathway, reduces cell death, and ameliorates symptoms in DSS-induced mice	Li et al. (2025)
Sodium butyrate	PINK1/Parkin	Sodium butyrate can promote mitophagy via AMPK activation and exerts protective effects on the intestinal epithelial barrier of IPEC-J2 cells under oxidative stress	Li et al. (2022)
Melatonin	PINK1/Parkin	Melatonin inhibits ROS-mediated PINK1/Parkin-dependent mitophagy and NF-κB/NLRP3-driven inflammation by activating Nrf2	Zhu et al. (2023)
Forsythiaside A	PINK1/Parkin	Forsythiaside A activates mitophagy and inhibits the NLRP3 inflammasome by mediating Nrf2 phosphorylation	Li et al. (2024), Liu et al. (2024a)
Urolithin A	PINK1/Parkin	Urolithin A enhances mitophagy by activating the PINK1-Parkin-mediated pathway. It also alleviates NLRP3/caspase-1-dependent inflammation	Fang et al. (2019), D’Amico et al. (2022)
Berberine	AMPK	Berberine reverses mitochondrial dysfunction caused by PINK1 deficiency through AMPK-dependent mitophagy	Um et al. (2023)
	HIF-1α/BNIP3	Berberine mediates BNIP3 expression by enhancing the binding of HIF-1α to the BNIP3 promoter, thereby promoting mitophagy and inhibiting apoptosis	Zhu et al. (2020)
Emodin	FUNDC1	Emodin promotes mitophagy through CK2-mediated dephosphorylation of FUNDC1, thereby inhibiting NLRP3 inflammasome activation	Fu et al. (2025)
Doxycycline	LC3B	Doxycycline induces mitophagy in IPEC-J2 cells, thereby inhibiting apoptosis through the removal of damaged mitochondria	Xing et al. (2017)
Metformin	LC3B	Metformin reduces ROS production by enhancing mitophagy, thereby decreasing the levels of pro-inflammatory cytokines IL-6 and IL-8 to alleviate inflammation	Toppila et al. (2024)
	AMPK/ULK1/PINK1/Parkin	Metformin activates the AMPK/ULK1/PINK1/Parkin mitophagy pathway, alleviating mitochondrial damage, and exerts a protective effect in DSS-induced colitis	Saber and El-Kader (2021), Guo et al. (2023)
Cannabidiol	PINK/Parkin	Cannabidiol stabilizes PINK1 on the mitochondrial membrane, recruits Parkin to damaged mitochondria, and simultaneously induces mitochondrial depolarization and ROS production	Ramirez et al. (2022)
MitoQ	Nrf2/PINK	Restoration of mitophagy via Nrf2-mediated regulation of PINK transcription and improvement of mitochondrial oxidative stress and abnormal mitochondrial dynamics	Xiao et al. (2016)
Rapamycin	mTORC1	Rapamycin induces mitophagy by directly binding to and inhibiting mTOR complex 1	Ling et al. (2023)
SS-31	PINK1	SS-31 effectively alleviates oxidative stress and inflammation through PINK1-mediated mitophagy	Ye et al. (2024)
*Lactobacillus* johnsonii JJB3	BNIPL/LC3B	sJJB3 alleviates intestinal oxidative stress-induced intestinal injury by modulating the gut microbiota and enhancing the BNIP3L-mediated mitophagy pathway	Liu et al. (2025b)
*Lactobacillus* casei ATCC 393	mTOR/PINK1	Synthetic selenium nanoparticles (SeNPs) can effectively alleviate H_2_O_2_-induced intestinal epithelial barrier dysfunction by modulating mTOR/PINK1-mediated mitophagy	Yan et al. (2021)

## 8 Conclusion and future perspectives

Mitophagy plays a fundamental role in maintaining intestinal homeostasis by preserving mitochondrial quality and regulating epithelial barrier integrity, immune cell function, and host-microbiota interactions. Increasing evidence indicates that dysregulated mitophagy contributes to IBD pathogenesis through mechanisms involving impaired epithelial cell function, altered immune responses, and mitochondrial dysfunction. In particular, genetic variants in autophagy-related genes such as *ATG16L1*, *NOD2*, and *IRGM* have been linked to defective mitochondrial quality control and increased susceptibility to intestinal inflammation. Recent studies highlight that therapeutic strategies aimed at restoring mitophagy, including natural bioactive compounds, synthetic pharmacological agents, and microbiota-based interventions, can improve mitochondrial function, reduce oxidative stress, and modulate inflammatory signaling in experimental models of IBD. These findings underscore the potential of mitophagy-targeted therapies as a novel approach for disease management. Future research should focus on elucidating the cell-type-specific regulatory mechanisms of mitophagy in intestinal epithelial cells and immune populations, as well as clarifying the interactions between mitophagy, host genetics, and the gut microbiome. Advances in multi-omics technologies and precision medicine approaches may further facilitate the identification of therapeutic targets and biomarkers associated with mitochondrial quality control. Collectively, targeting mitophagy represents a promising direction for the development of next-generation, mechanism-based therapeutic strategies for IBD.

## Glossary

IBD: Inflammatory bowel disease
UC: Ulcerative colitis
CD: Crohn’s disease
ATP: Adenosine triphosphate
OXPHOS: Mitochondrial oxidative phosphorylation
ROS: Reactive oxygen species
PINK1: PTEN-induced kinase 1
PARL: Presenilin associated rhomboid like
IMM: Inner mitochondrial membrane
OMM: Outer mitochondrial membrane
ER: Endoplasmic reticulum
SQSTM1/p62: Sequestosome 1
MAP1LC3/LC3: Microtubule-associated protein 1 light chain 3
LIR: LC3-interacting region
BNIP3: BCL2 interacting protein 3
BNIP3L/NIX: BCL2/adenovirus E1B 19-kDa-interacting protein 3-like
FUNDC1: FUN14 domain containing 1
GWAS: Genome-wide association studies
SNPs: Single-nucleotide polymorphisms
*NOD2*: nucleotide-binding oligomerization domain-containing protein 2
*ATG16L1*: autophagy-related protein 16-like 1
*IRGM*: immunity-related GTPase family M
IECs: Intestinal epithelial cells
TJs: Tight junctions
AJs: Adherens junctions
ZO-1: Zonula occludens-1
JAMs: Junctional adhesion molecules
ISCs: Intestinal stem cells
MUC: Mucin
DCs: Dendritic cells
DAMPs: Damage-associated molecular patterns
PAMPs: Pathogen-associated molecular patterns
TLR: Toll-like receptors
NF-κB: Nuclear factor-kappaB
GSDMD: Gasdermin-D
MHC-II: Major histocompatibility complex class II
Treg: Regulatory T cell
